# The complete mitochondrial genome of *Taeniothrips tigris* Bhatti, 1995 (Thysanoptera: Thripidae)

**DOI:** 10.1080/23802359.2021.1947916

**Published:** 2021-07-06

**Authors:** Avas Pakrashi, Kaomud Tyagi, Vikas Kumar

**Affiliations:** a Centre for DNA Taxonomy, Molecular Systematics Division, Zoological Survey of India, Kolkata, India; b Department of Zoology, University of Calcutta, Kolkata, India

**Keywords:** Mitogenome, Thripidae, *Thrips*

## Abstract

In this study, we sequenced complete mitogenome of *Taeniothrips tigris* Bhatti 1995. It was 15,501 bp in length containing 13 protein-coding genes, 22 transfer RNA genes, and 2 ribosomal RNA genes along with two non-coding regions. The overall base composition of *Ta. tigris* is 43.66% A, 35.20% T, 11.46% C, and 9.68% G, with a high AT bias of 78.86%. The constructed phylogeny using 19 mitogenomes revealed that the genus *Taeniothrips* is in close relationship with genus *Thrips*. This mitogenome data would help in deducing phylogenetic relationships studies in the order Thysanoptera.

The members of the order Thysanoptera (*Thrips*) are minute, soft-bodied, and can be distinguished from the other insects by the presence of fringed wings and asymmetrical mouthparts (Mound and Marullo 1996). Out of 6200 known species, only 1% of thrips are reported as a serious pest (Thripswiki 2021). They are the sole transmitter of plant Tospoviruses, causing severe economic damage to a wide number of agricultural and horticultural crops (Riley et al. 2011; Tyagi et al. 2017). The genus *Taeniothrips* was first described by Amyot and Serville (1843) with Type species *Thrips primulae* Haliday. Later on Bhatti (1995) considered the *Taeniothrips tigris* as a type species of the genus *Taeniothrips* (*Fetothrips*). *Ta. tigris* is an endemic species to India and belongs to the most diverse subfamily Thripinae of the family Thripidae. This is the first report of mitogenome data for genus *Taeniothrips* in the order Thysanoptera.

The specimens of *Ta. tigris* were collected by a bush beating method in March 2020 at Nainital (29.38N, 79.45E), Uttarakhand, India. DNA was extracted by a nondestructive method (Tyagi et al. 2017) with DNeasy Blood and Tissue Kit (QIAGEN, Hilden, Germany -). All voucher specimens (registration No. 11077/H17-11104/H17) were deposited in the National Zoological Collections (NZC) at the Center for DNA Taxonomy, Molecular Systematics Division, Zoological Survey of India, Kolkata. The sequence data of *Ta*. *tigris* was generated on the Illumina platform NovaSeq 6000 (Illumina, San Diego, CA), with 2 × 150 base pair’s chemistry. Assembly was done by GetOrganelle software version 1.7.4 (Jin et al. 2020) and annotated in MITOS Web Server (Bernt et al. 2013), ORF Finder (https://www.ncbi.nlm.nih.gov/orffinder), BLASTn, and BLASTp and further compared with the thrips mitogenomes available on GenBank.

*Ta. tigris* mitogenome was 15,501 bp in length with 37 genes, including 13 protein-coding genes (PCGs), 22 transfer RNAs (tRNAs), 2 ribosomal RNAs (rRNAs), and 2 putative control regions (Figure S1). Most of the genes were located on the majority strand except *nad5*, *nad4*, *nad4L, trnH*, *trnP*, and *trnY*. The AT content of the genome was 78.86% (43.66% of A, 35.20% of T) and GC 21.14% (9.68% of G, 11.46% of C). ATA start codon was used by *cox2, cox3, cytb, nad2, nad4, nad5, atp8*; ATT by *cox1, nad1, nad3, nad6*, *atp6*; and ATG by *nad4L*. All the PCGs were stopped with TAA stop codon with few exceptions. TAG stop codon was used by *atp6*, *cox1;* and incomplete termination codon T(AA) was used by *nad2*, *nad3, nad4*, and *atp8*. All the tRNAs had typical cloverleaf secondary structure with the length ranging from 71 bp (*trnI*) to 51 bp (*trnV*). The length of *rrnL* and *rrnS* were 1222 and 757 bp, respectively. *Ta. tigris* mitogenome contained 7 overlapping regions (1–7 bp with a total of 24 bp) and 21 intergenic spacer regions (1–165 bp with a total of 841 bp).

**Figure 1.**
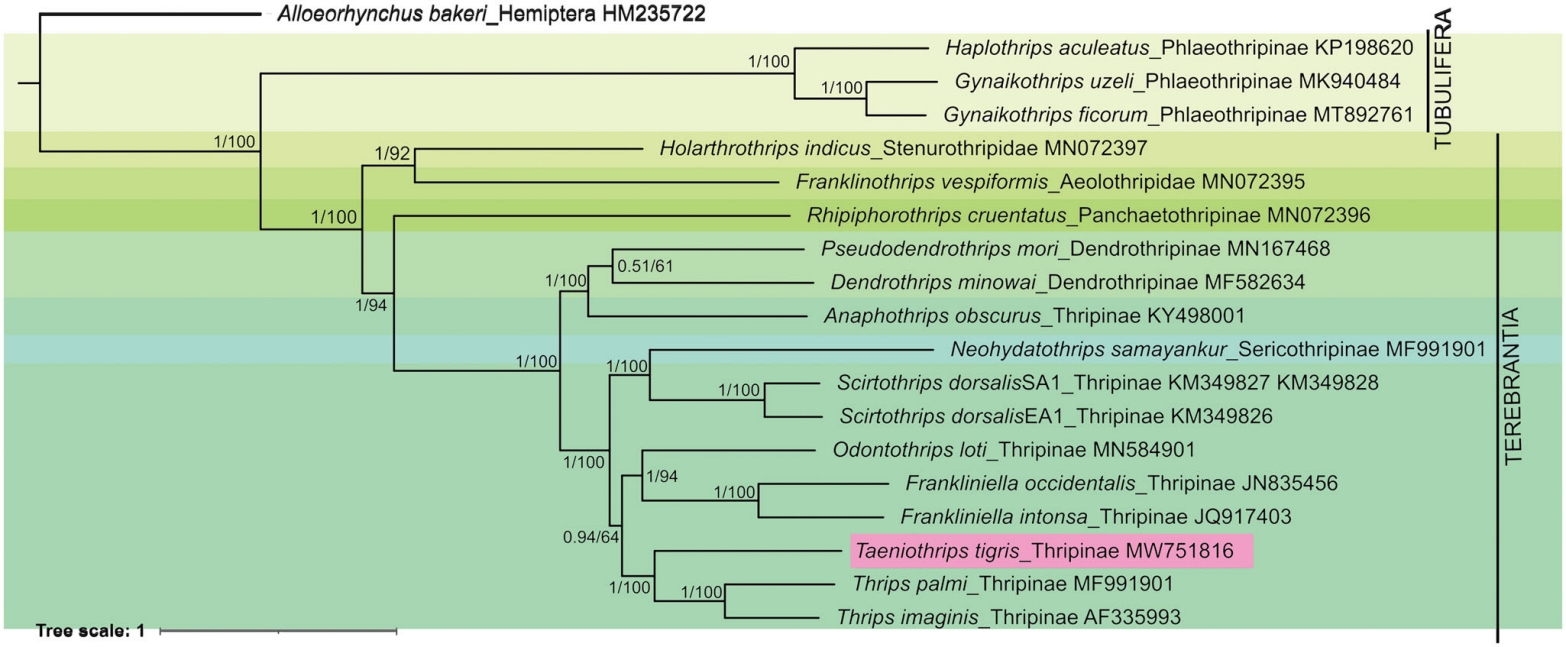
Phylogenetic tree inferred from 13 PCGs using Bayesian Inference. The posterior probabilities (pp) and bootstrap support (bs) are marked beside the nodes. The GenBank accession numbers are also provided. The hemipteran, *Alloeorhynchus bakeri* was used as an outgroup.

The PCGs were aligned with the MAFFT algorithm in TranslatorX tool (Abascal et al. 2010) and concatenated using SequenceMatrix version 1.8 (Vaidya et al. 2011). The best fit model was detected as ‘GTR + I + G’ in PartitionFinder version 2.1.1 using BIC criterion (Lanfear et al. 2017). The Bayesian inference (BI) and maximum likelihood (ML) phylogenetic trees were constructed using Mr. Bayes version 3.2 (Ronquist et al. 2012) and IQ tree web server (http://iqtree.cibiv.univie.ac.at/), respectively, with *Alloeorhynchus bakeri* (Genbank accession HM235722) as an outgroup. Both the phylogenetic methods produced similar tree topology and superimposed as shown in Figure 1. The phylogeny revealed *Ta. tigris* was closely related to the genus *Thrips* (*Th*. *palmi* and *Ta*. *imaginis*) in subfamily Thripinae. The close relationship of the genus *Taeniothrips* and *Thrips* is also evidenced by the morphological characters as they do not have ocellar setae I. In contrast, the ctenidium on lateral side of tergites is present in *Thrips* and absent in *Taeniothrips* (Bhatti 1980; Mound et al. 2012). The complete mitogenome data of *Ta. tigris* will further broaden the knowledge gap in understanding the evolution, phylogeny, and gene arrangement in order Thysanoptera.

Acknowledgments

The authors are thankful to the Director of Zoological Survey of India (ZSI), Ministry of Environment, Forests and Climate Change (MoEFCC), Govt. of India for providing necessary permissions and facilities. This work is part of Ph.D. thesis of Avas Pakrashi.

## Data Availability

The data that support the findings of this study are openly available in GenBank of NCBI at https://www.ncbi.nlm.nih.gov, reference number MW751816.
